# Identification of stromal ColXα1 and tumor-infiltrating lymphocytes as putative predictive markers of neoadjuvant therapy in estrogen receptor-positive/HER2-positive breast cancer

**DOI:** 10.1186/s12885-016-2302-5

**Published:** 2016-04-18

**Authors:** Alexander S. Brodsky, Jinjun Xiong, Dongfang Yang, Christoph Schorl, Mary Anne Fenton, Theresa A. Graves, William M. Sikov, Murray B. Resnick, Yihong Wang

**Affiliations:** Department of Pathology and Laboratory Medicine, Rhode Island Hospital and Lifespan Medical Center, Warren Alpert Medical School of Brown University, Providence, USA; Department of Pathology, Women and Infants Hospital, Warren Alpert Medical School of Brown University, Providence, USA; Molecular Biology, Cell Biology, & Biochemistry, Brown University, Providence, USA; Department of Medicine, Rhode Island Hospital and Lifespan Medical Center, Warren Alpert Medical School of Brown University, Providence, USA; Department of Surgery, Rhode Island Hospital and Lifespan Medical Center, Warren Alpert Medical School of Brown University, Providence, USA; Program in Women’s Oncology, Women and Infants Hospital, Warren Alpert Medical School of Brown University, Providence, USA; Department of Pathology, Rhode Island Hospital and Lifespan Medical Center, Providence, RI 02903 USA

**Keywords:** Collagen, Tumor microenvironment, HER2-positive breast cancer, Neoadjuvant chemotherapy, Tumor infiltrating lymphocytes

## Abstract

**Background:**

The influence of the tumor microenvironment and tumor-stromal interactions on the heterogeneity of response within breast cancer subtypes have just begun to be explored. This study focuses on patients with estrogen receptor-positive/human epidermal growth factor receptor 2-positive (ER+/HER2+) breast cancer receiving neoadjuvant chemotherapy and HER2-targeted therapy (NAC+H), and was designed to identify novel predictive biomarkers by combining gene expression analysis and immunohistochemistry with pathologic response.

**Methods:**

We performed gene expression profiling on pre-NAC+H tumor samples from responding (no or minimal residual disease at surgery) and non-responding patients. Gene set enrichment analysis identified potentially relevant pathways, and immunohistochemical staining of pre-treatment biopsies was used to measure protein levels of those pathways, which were correlated with pathologic response in both univariate and multivariate analysis.

**Results:**

Increased expression of genes encoding for stromal collagens, including Col10A1, and reduced expression of immune-associated genes, reflecting lower levels of total tumor-infiltrating lymphocytes (TILs), were strongly associated with poor pathologic response. Lower TILs in tumor biopsies correlated with reduced likelihood of achieving an optimal pathologic response, but increased expression of the Col10A1 gene product, colXα1, had greater predictive value than stromal abundance for poor response (OR = 18.9, *p* = 0.003), and the combination of increased colXα1 expression and low TILs was significantly associated with poor response in multivariate analysis. ROC analysis suggests strong specificity and sensitivity for this combination in predicting treatment response.

**Conclusions:**

Increased expression of stromal colXα1 and low TILs correlate with poor pathologic response in ER+/HER2+ breast tumors. Further studies are needed to confirm their predictive value and impact on long-term outcomes, and to determine whether this collagen exerts a protective effect on the cancer cells or simply reflects other factors within the tumor microenvironment.

**Electronic supplementary material:**

The online version of this article (doi:10.1186/s12885-016-2302-5) contains supplementary material, which is available to authorized users.

## Background

Breast cancer treatment is largely determined by hormone receptor and human epidermal growth factor receptor-2 (HER2) expression, but there is significant variability of response and prognosis within the subtypes defined by these markers. Being able to identify characteristics or markers on pretreatment samples that predict a higher likelihood of treatment-refractory disease could spare patients from exposure to ineffective and often toxic therapies, and promote the development of novel treatments that target and neutralize these factors.

In the past, most analyses have focused on identifying markers expressed by the tumor cells themselves. Tumor biopsies and surgical specimens consist of a mixture of cancer cells and surrounding stroma comprised of a variety of cell types, and while the traditional approach to tumor biology disregarded the impact that those other tissues might have on tumor behavior, more recently there has been an increased appreciation of the possibility that the tumor microenvironment and tumor-stromal interactions could play an important role in determining response. These include the abundance and character of tumor-infiltrating lymphocytes (TILs) and levels of expression of proteins such as PD-L1 that can modify immune response to the growing tumor, both of which may have a significant impact on prognosis, especially in more aggressive breast cancer subtypes [1, 2].

Response rates to NAC vary widely depending on subtype in breast cancer. In HER2+ patients, the addition of the HER2-targeting monoclonal antibody trastuzumab to standard NAC has been shown to improve not only the pathological Complete Response (pCR) rate in the NAC setting, but also recurrence-free and overall survival in the adjuvant setting [3]. However, despite the addition of trastuzumab or even dual HER2-targeting therapies with trastuzumab and either lapatinib or pertuzumab to NAC (NAC+H), a significant percentage of HER2+ patients do not achieve a pCR or minimal residual disease. In the TRYPHAENA trial, the pCR for ER-/HER2+ is 77 % but only 48 % for ER+/Her2+ with cases from all three arms combined [4]. In NeoSphere trials, few pathologic complete responses were noted in tumors that are hormonal receptor positive in all four arms [5]. There was a significant difference in pCR rates between hormone positive and negative tumors; the pCR was 40 % for ER- groups and only 17 % for ER+ groups. This discrepancy may reflect differences in cancer cell biology, related to proliferation or dependence on HER2-mediated signaling; it is also possible that differences in the microenvironment mediate response. At this time, there is no reliable, validated method to distinguish responders from non-responders. Thus, many patients are needlessly treated with toxic chemotherapy with uncertain benefit from the treatment. The aim of this work was to examine gene expression profiles of tumors at the time of the pre-treatment biopsy to identify molecular features that may be associated with chemoresponse. Such markers of NAC response could be useful to reduce chemotherapy-reduced morbidity, and identify new therapeutic approaches to treat ER+/HER2+ breast cancer.

In this study, we chose to focus on the ER+/HER2+ subtype where patients do not respond well to NAC and suffer from overall survival rates comparable to TNBC. The cross-talk between these oncogenic pathways drives cross-resistance to current therapies, leading to the use of cytotoxic chemotherapy to treat this subtype [6]. We hypothesize that new predictive markers could be developed by identifying candidate genes and pathways from gene expression profiling followed by detailed analysis of candidate markers using immunohistochemistry [7, 8]. We found that collagens, in particular the expression of the protein product from the ColXA1 gene, were strongly associated with NAC response in ER+/HER2+ breast tumors. The presence of collagen in the surrounding tumor milieu has long been known to influence cancer cells. Collagens can induce epithelial-mesenchymal transitions and related invasive properties of breast cancer cells [9]. However, the utility of any specific collagen as a prognostic marker remains unclear. Expression of ColXA1 has been included in published stromal expression signatures [10, 11], but the expression of ColXA1 protein product, colXα1, has not been evaluated. For these reasons, we examined the potential for the expression of the colXα1 protein by immunohistochemistry to predict response to NAC in ER+/HER2+ breast tumors.

## Methods

### Patients and tissue samples

Selection of patients and analysis was approved by the Rhode Island Hospital Institutional Review Board, approval #467617, and the Women and Infants Hospital Institutional Review Board, #14–0090. Written informed consent was obtained from each patient for tissue collection. A retrospective natural language search of the surgical pathology databases was performed to identify all patients who received NAC. Among patients who received NAC at the Lifespan Comprehensive Cancer Centers at Rhode Island Hospital and Miriam Hospital or at Women and Infants Hospital of Rhode Island between 2007 and 2014, we identified those with ER+/HER2+ cancers who received NAC+H and for whom sufficient tissue was available for analysis (Table 1, see Additional file 1: Table S1). The biopsy samples in some cases were exhausted after multiple immunohistochemistry and florescent in situ hybridization studies, which could not be included in this study. H&E slides of all biopsies were reviewed. We reviewed histological features such as tumor type, size, extent of the disease, lymph node status and histological grade using the Nottingham combined histologic grading system. ER/PR/Her2 staining was the data retrieved from the pathology reports for the purpose of the study. HER2 was considered to be positive if the grade of immunostaining was 3+, or a 2+ result showed gene amplification via fluorescent in situ hybridization (FISH). In the FISH analyses, each copy of the HER2 gene and its centromere 17 (CEP17) reference were counted. The interpretation followed the criteria of the ASCO/CAP guidelines for HER2 IHC classification for breast cancer: positive if the HER2/CEP17 ratio was higher than 2.0 [12].

**Table 1:** Association of clinical characteristics to neoadjuvant treatment response by subtype

Characteristic	No.	% Good Response	*P*
ER+/HER2+ cases used for Collagen X IHC			
No. of patients	50	36	
RCB			
0	8		
1	10		
2	6		
3	26		
Age, y			0.006
<50	18	62	
≥50	32	28	
Pre-Treatment Lymph Node Status			0.3
Negative	15	47	
Positive	35	31	
Pre-treatment Tumor Stage			0.8
T1c/T2	35	37	
T3/T4	15	33	
Tumor Grade			0.35
2	21	28.6	
3	29	41.4	
colXα1			0.000^a^
0	9	87.5	
1	17	58.8	
2	9	0	
3	15	0	
sTIL			0.007^a^
0 ≤ 10 %	20	15	
11 ≤ 20 %	9	22	
21 ≤ 30 %	5	40	
31 ≤ 40 %	7	42	
41 ≤ 50 %	7	86	
51 ≤ 60 %	2	100	
>60 %	0		
Stroma			0.005^a^
0	2	100	
1	23	52	
2	25	16	
All ER+/Her2+ Cases			
No. of patients	74	41	
RCB			N/A
0	14		
I	16		
II	11		
III	33		
Age, y			
<50	35	51	0.07
≥50	39	31	
Pre-Treatment Lymph Node Status			0.47
Negative	26	46	
Positive	48	38	
Tumor Grade			0.33
2	25	32	
3	49	45	
Pre-treatment Tumor Stage			0.09
T1c/T2	51	47	
T3/T4	23	26	
sTIL			0.000^a^
0 ≤ 10 %	27	11	
11 ≤ 20 %	15	40	
21 ≤ 30 %	8	37.5	
31 ≤ 40 %	10	60	
41 ≤ 50 %	12	83	
51 ≤ 60 %	2	100	
>60 %	0		
Stroma			0.000^a^
0	6	100	
1	39	51.3	
2	29	14.0	

*P* was calculated by Fisher exact test. ^a^ Pearson Chi-Square *p*-value

Pathological response to NAC was assessed by the AJCC cancer staging and residual cancer burden (RCB) score after 3–6 months of treatment [13]. The RCB system stratifies patients with residual invasive cancer by size and invasive cellularity of the residual tumor bed, number of involved lymph nodes and largest focus of cancer in an involved node into classes I, II, and III (RCB class 0 is synonymous with having achieved a pCR), which has been shown to correlate with distant breast cancer recurrence in patient with HER2+ cancers [14]; on-line calculator available at http://www.mdanderson.org/breastcancer_RCB. Patients who achieved a pCR or minimal residual disease (RCB class 0 and I) were considered good pathologic responders, while patients with more significant residual disease (RCB class II-III) were considered poor pathologic responders. Cases from 2007, before RCB guidelines were first were reviewed and RCB scores were calculated, were based on information from pathology reports. Two of the 74 patients received hormonal therapy. Five cases receiving chemotherapy of unknown type were included in this study because the study examined a range of different neoadjuvant therapies and these patients were verified to have received chemotherapy (see Additional file 1: Table S1). The observations are therefore contingent on receiving neoadjuvant chemotherapy for ER^+^/HER2^+^ breast tumors.

### Microarray and qPCR analysis

#### RNA extraction and purification

From the ER+/HER2+ patients we selected a mixture of good and poor responders for whom we had sufficient tissue for this assay. Ten micron tumor sections were scraped from the slides for total RNA extraction. RNA was purified using the RecoverAll Total Nucleic Acid Extraction Kits for FFPE tissues (Ambion, Austin, TX) and further purified and concentrated with the RNEasy Minelute Cleanup Kit (Qiagen, Valencia, CA).

#### Expression microarray and qPCR

RNA was isolated and purified using the RNeasy FFPE kit (Qiagen, Valencia, CA, USA). One hundred nanograms of total RNA was amplified using Affymetrix’ Sensation Plus FFPE amplification kit following the manufacturer’s instructions and labeled cDNA was hybridized to Affymetrix (Santa Clara, CA, USA) HTA 2.0 microarrays and visualized at the Brown University Genomics Core Facility following the manufacturer’s instructions. Signals were estimated using RMA [15]. Fold change, t-tests, and multiple hypothesis tests were calculated in R. Data are available in GEO, GSE67982.

For real-time qPCR, cDNA was prepared using QuantiTect Reverse Transcription Kit (Qiagen). qPCR was performed on a Mx3005p (Agilent) with Brilliant III SYBR Green (Agilent). Relative expression fold changes were calculated relative to GAPDH.

#### Gene expression and pathway analysis

Microarray signals were analyzed for statistical significance in terms of differences between samples between good and poor responders. We applied gene set enrichment analysis (GSEA) to investigate pathways and groups of genes that may be associated with NAC response, which identified collagens and immune pathways as strongly associated with good pathologic response. The collagen transcript, Col10A1, was one of the top-ranked transcripts associated with NAC response for which an available commercial antibody was available. Collagen, type 10, alpha 1 (gene name Col10A1 and protein product ColXα1) is a secreted, homotrimeric short-chain collagen and is up-regulated in a variety of tumor types with restricted or undetectable expression in a large spectrum of normal tissues, normal primary cultures and tumor cell lines [7, 8]. After verification of the microarray observations by qPCR, we tested the association of ColXα1 expression as well as other tumor microenvironmental factors such as the abundance of tumor associated stroma and TILs in the pre-treatment biopsy samples to correlate with post-treatment response. TCGA RNA-seq data for breast invasive carcinoma were downloaded from the Firehose Broad GDAC [16]. TCGA clinical data were downloaded from the TCGA data archive in September 2015 (http://cancergenome.nih.gov/).

#### Tumor-associated stroma and TIL analysis

We morphologically evaluated the amount intratumoral stroma and TILs on pre-treated biopsy samples which commonly consisted of 2–5 needle cores of average 1.5 cm in length obtained with either a 14 gauge spring-loaded biopsy device or a 12 gauge vacuum-assisted biopsy device. The amount of intratumoral stroma was scored as 0 to 2: 0 for absent or minimal stroma (<10 %), 1 for mild to moderate amount of stroma (10–40 %) and 2 for abundant stroma (≥40 %). Stromal and intratumoral TILs (sTILs and iTILs) were evaluated based on criteria published by Denkert et al. [2]. Briefly, iTILs were defined as lymphocytes in direct contact with the tumor cells, whereas sTILs were defined as lymphocytes in the surrounding stroma, with the percent of the tumor or stromal volume comprised of infiltrating lymphocytes, as opposed to tumor or other stromal tissues, on an H&E stained biopsy section estimated by the reading pathologists, with results reported in increments of 10 (0–1 % was scored as 0, with all other estimates rounded up to the next highest decile - i.e., 11–20 % was scored as 20). sTILs and iTILs were totaled to calculate TILs. The trends were similar for each lymphocyte fraction (data not shown). sTILs were chosen to be analyzed as they were considered to the most consistent metric as recommended by the International TILs Working Group [17]. The histological evaluation was graded independently by two pathologists (YW and JX), who were blinded to clinical information including the post-treatment outcome, at the time of analysis, with the summary score representing the mean of the two separate scores. The two pathologists evaluated 30 separate cases (triple negative breast cancer cases) together to get a general agreement of the sTIL. The actual study cases were evaluated independently, the concordance is about 95 %. The cases with greater than 10 % difference were reviewed together and the average score was used.

#### Immunohistochemistry and ColXα1 expression scoring

Four-micron sections were cut from formalin-fixed paraffin-embedded tissue blocks, heated at 60 °C for 30 min, deparaffinized, rehydrated and subjected to antigen retrieval by heating the slides in epitope retrieval buffer in a water bath at 95 °C for 45 min. The slides were then incubated with either mouse monoclonal antibodies or rabbit polyclonal antibodies for 30 min at room temperature in a DAKO Autostainer. Anti- colXα1 (1:50, eBioscience/Affymetrix, Clone X53), estrogen receptor (1:50, DAKO (Santa Clara, CA, USA), clone 1D5), progesterone receptor (1:400, DAKO, clone 1A6), and HER2/neu (DAKO HercepTest^TM^) were used for immunohistochemistry. The immunoreactivity was detected using the DAKO EnVision method according to the manufacturers recommended protocol. Peri- and intra-tumoral stromal staining for ColXα1 was scored as 0, 1+, 2+, and 3+. Briefly, 0 as no staining; 1+ as weak staining; 2+ as <10 % of stroma tissue with intense staining present; 3+ as >10 % of stroma tissue with patchy intense staining. All scoring was performed blinded to the outcome and many cases were scored before the outcome data was available.

#### Statistical analysis

SPSS v22 for MacOS (SPSS, Chicago, IL, USA) was used for all statistical analysis. *P* < 0.05 was considered statistically significant. All *p*-values reported are two-sided. For the logistic regression, all factors were analyzed as continuous variables.

## Results

### Patients and clinical information

**Fig. 1:**
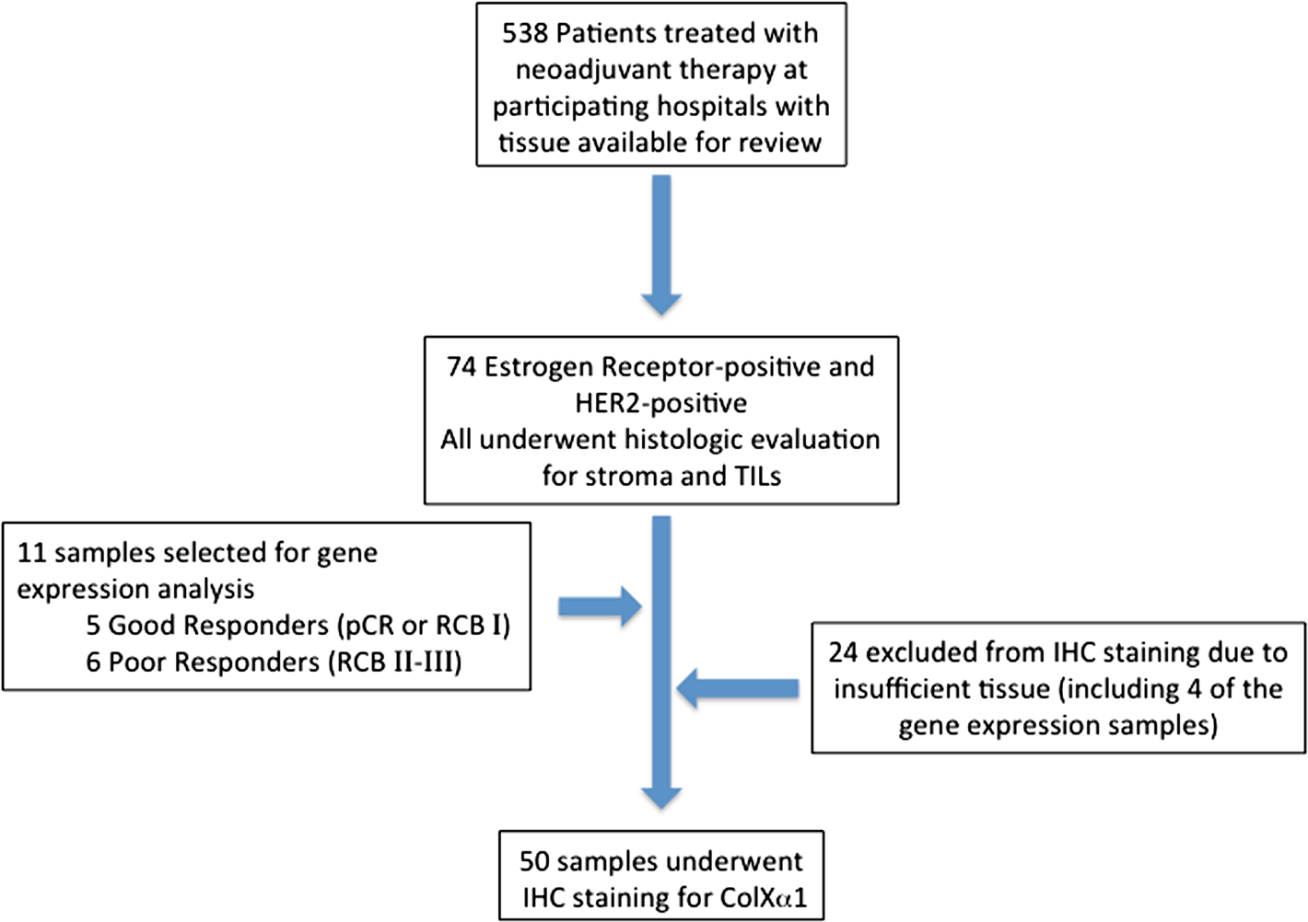
Flow diagram of the approach to identify and test Col10A1 in ER+/HER2+ breast tumors. From 538 patients, 74 ER+/HER2+ breast tumors were selected for analysis. 11 ER+/HER2+ tumors were selected for expression profiling using Affymetrix HTA 2.0 microarrays. After qPCR verification, we evaluated the level of colXα1 protein in primary tumors before NAC using immunohistochemistry to test the expression of colXα1 protein levels in 50 ER+/HER2+ breast tumors with available tissue

Among 538 patients who received NAC at the participating hospital, we identified 74 ER+/HER2+ patients for whom we had pathologic response data and sufficient pretreatment tissue for analysis (Fig. 1). Their clinical and pathologic data are summarized in Table 1; 92 % (68 of 74) were clinical stage ≥ IIB. Most received either doxorubicin and cyclophosphamide followed by paclitaxel and trastuzumab (*n* = 33) or docetaxel, carboplatin and trastuzumab (*n* = 35). The addition of pertuzumab to the neoadjuvant regimen for HER2+ cancer had not yet been routinely adopted. 19 % (14 of 74) of patients achieved a complete pCR (RCB class 0), and 40.54 % (30 of 74) had a good pathologic response (RCB class 0 or I). There were no significant statistical differences in the post-treatment response between patients who received TCH vs. AC-TH treatment options.

### Association of Col10A1 mRNA and NAC response in ER+/HER2+ cancer

In order to identify novel markers for NAC response in this subtype, we randomly selected 5 tumors sampled from patients who achieved good response (RCB 0 or I) with NAC+H and 6 from patients who did not achieve a good response (RCB II or III) for genome-wide expression profiling using Affymetrix HTA 2.0 microarrays (Fig. 1). We hypothesized that even with a small set of cases, candidate markers strongly associated with pCR would be detected. Only 30 transcripts were significantly differentially expressed (Fc >2, *p* < 0.05) including three collagens (subtypes Col10A1, Col14A1, and Col3A1), which were up-regulated in tumors that had poor response (see Additional file 2: Table S2). Other differentially expressed genes associated with more aggressive breast cancer including ERBB4 and TGFB3 are up-regulated in poor responders in these data. However, qPCR analysis of TGFB3 in 42 tumors did not find a strong association with response [18]. Likely because of the small number of significantly differentially expressed transcripts, no transcripts had a corrected *p* < 0.05 after multiple hypothesis correction. Because so few genes were considered significantly differentially expressed, representative significantly differentially expressed genes were verified by qPCR (see Additional file 3: Figure S1).

We performed pathway analysis to identify groups of genes associated with good response. Gene Set Enrichment Analysis (GSEA) identified many pathways significantly biased towards either good responders or resistant tumors (see Additional file 4: Table S3). In ER+/HER2+ tumors, within the Gene Ontology gene sets, increased expression of immune pathways, and components of the cell cycle were associated with pCR, while drug metabolism, RNA metabolism, and expression of certain collagens were associated with poor responding tumors (see Additional file 4: Table S3).

**Fig. 2:**
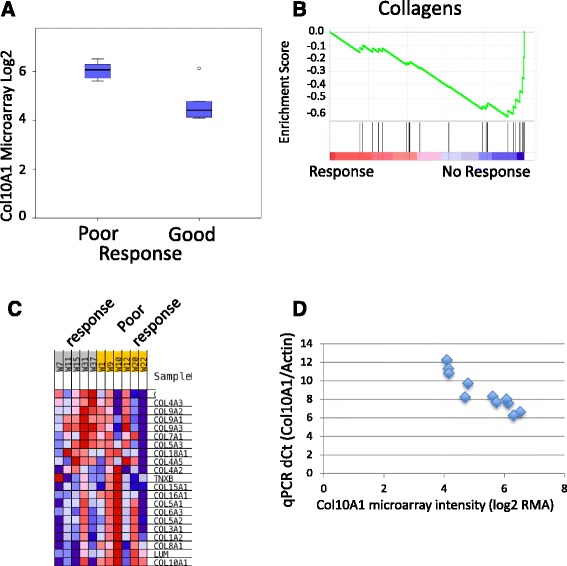
Association of colXα1 expression with NAC response. **a** Box plot of the Col10A1 probeset on the Affymetrix HTA 2.0 microarray distinguishes good and poor responding ER+/HER2+ breast tumors. The one outlier on the array, has an intermediate colXα1 IHC score of 1. **b** Gene Set Enrichment Analysis reveals enrichment of the Gene Ontology (GO) category, collagens, in pCR resistant ER+/HER2+ breast tumors. Each black line represents one gene in the GO collagen gene set. **c** Heat map of mRNA expression changes for all measured collagens on the microarray. **d** qPCR of Col10A1 mRNA expression correlates with the microarray data

We aimed to identify a representative transcript of a pathway or group of transcripts that we could test by IHC in an extended cohort of tumors. The collagen Gene Ontology gene set is strongly biased towards poor responding tumors (NES = −1.9, FDR = 0.009) (Fig. 2) and three transcripts encoding collagens (Col10A1, Col14A1, and COL3A1) were among the most significant differentially expressed genes (see Additional file 4: Table S3).

To validate the microarray observations, we performed qPCR on five transcripts, significantly differentially expressed (Fc > 2, *p* < 0.05) between responding and non-responding tumors among those analyzed by microarrays and found good overall correlation (*R* = 0.69, *P* <0.001), (See Additional file 3), including the Col10A1 transcript (Fig. 2d).

### Total tumor-infiltrating lymphocytes and tumor-associated stroma are associated with good response in ER+/HER2+ tumors

The gene expression data (Additional file 2: Table S2 and Additional file 4: Table S3) suggested that higher levels of lymphocytes were associated with achieving a good response. This is highlighted by increased expression of CXCL10 (Fc 1.8, *p* = 0.01) and IL7R, highly ranked by GSEA, in responsive tumors. These gene expression data predicted that examination of infiltrating lymphocytes is warranted and that such TILs would be associated with achieving good response.

To test the gene expression observations, we examined each tumor for the number of TILs. TILs have been proposed as a predictor of pCR in TNBC [19]. However, the association between TILs and good responders in ER+/HER2+ tumors remains uncertain. We found that higher levels of TILs corresponded to tumors with good responders in the full 74 ER+/HER2+ patient cohort (Table 1). In univariate analysis using a logistic regression model, TILs were found to be predictive for good response (OR = 0.94, *P* = 0.001) (Table 2), and the association with good response was observed for both tumor-associated stroma and TILs (Table 1 and Additional file 5: Table S4).

**Table 2:** Odds of response after neoadjuvant chemotherapy from logistic regression model. *N* = 50

Characteristic	OR (95 % CI)	*P*
Univariate		
Age	5.6 (1.6–20.0)	0.008
sTIL	0.46 (0.29–0.72)	0.001
Stroma	6.6 (1.9–23.4)	0.003
colXα1	18.9 (2.8–129)	0.003
Multivariate		
Age	0.23 (0.009–5.9)	0.37
sTIL	0.39 (0.16–0.92)	0.03
Stroma	1.9 (0.17–22)	0.6
colXα1	28 (1.6–487)	0.022

### ColXα1 expression predicts response to NAC in ER+/HER2+ cancer

Col10A1 was the most significantly biased collagen in the GSEA analysis (Fig. 2c). COL10A1 has been included in stromal expression signatures in breast cancer [10]. Therefore, the protein product of the Col10A1 gene, colXα1 was a strong candidate to predict NAC response in ER+/HER2+ breast tumors and warranted further evaluation at the protein level based on the literature and these gene expression data.

**Fig. 3:**
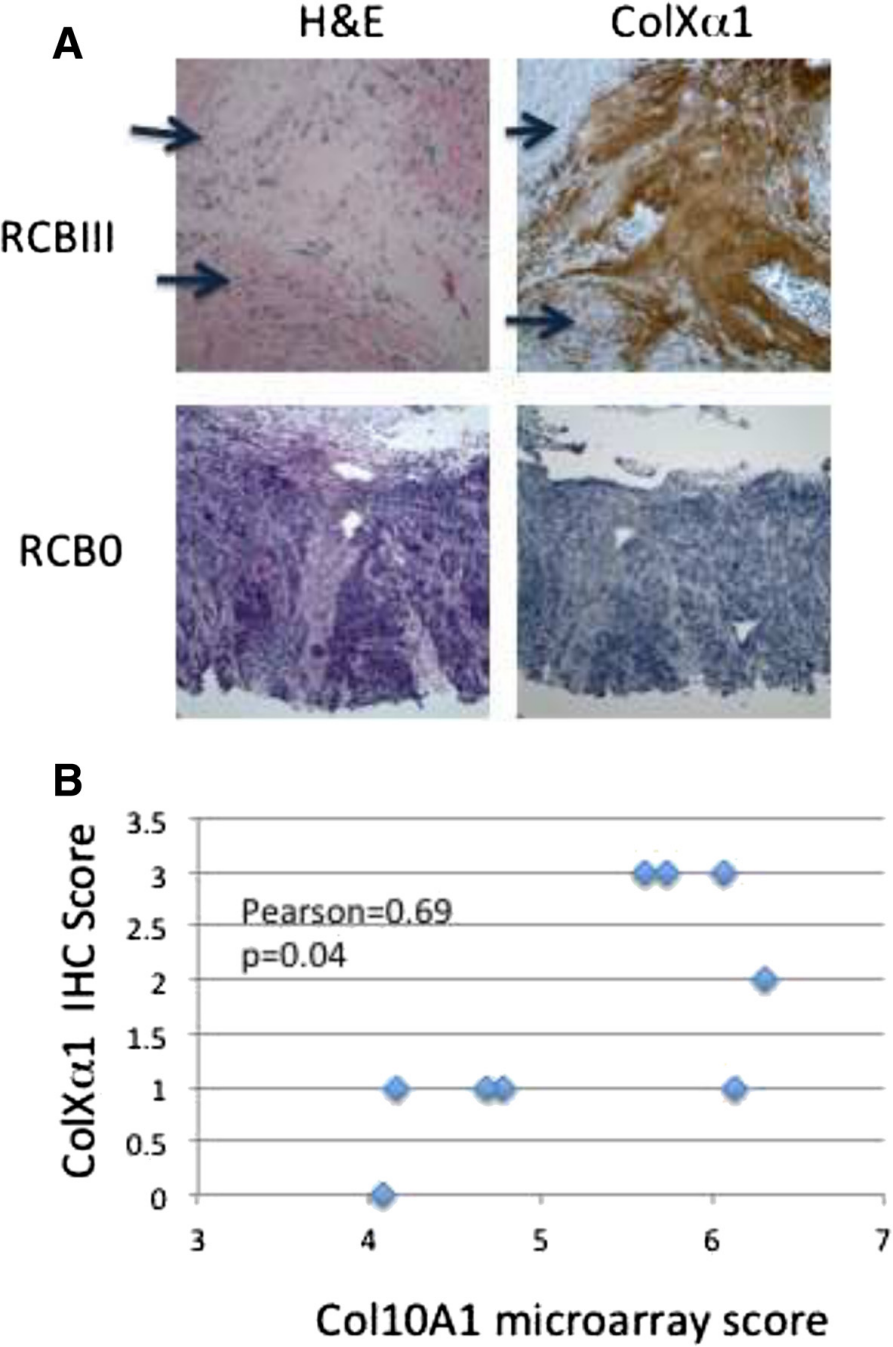
Immunohistochemistry of colXα1. **a** Representative colXα1 immunostaining in low- and high- colXα1 expressing ER+/HER2+ breast cancers. Two representative cases, one with no response, RCBIII, and strong colXα1 signal, score = 2, and one with good response, RCB0, and no colXα1 signal, score = 0, are shown. Arrows indicate regions with tumor cells. **b** RNA levels as determined by the microarray correlate with colXα1 IHC signal in 9 cases

To evaluate the findings from the gene expression data that collagens are significantly associated with pCR, we tested the usefulness of an anti-colXα1 monoclonal antibody to predict poor response and evaluated its relationship with other microenvironment metrics including the amount of tumor-associated stroma and TILs for its role in pCR. We performed IHC in 10 reduction mammoplasty cases to define the colXα1 expression pattern in normal breast tissue. In normal breast tissue, stain was negative for colXα1 except for occasional faint staining in a perivascular distribution pattern (data not shown). Among the 74 ER+/HER2+ cases in our study group, 50 pre-treatment needle biopsy samples had sufficient residual material (at least 1 cm tumor/stroma in a 12 gauge needle core) to allow evaluation with anti- colXα1 IHC. The overall response rate (pCR + RCB I) in this set was 36 % (18 of 50 patients) Table 1. Microenvironmental factors including decreased amount of stroma (*P* = 0.016) and higher levels of TIL (*P* < 0.001) were associated with good response in these 50 cases (Table 1). In tumor samples, immunostaining of colXα1 was observed as intense peri- and intra-tumoral distribution in some tumors in the RCBIII case (Also see 20× image in Additional file 6: Figure S2). A periductal/perivascular colXα1 staining pattern was frequently observed (Fig. 3). Increased colXα1 staining was strongly associated with a poor response by a chi-squared test (*P* < 0.001) (Table 1). The two cases with no stroma were scored as having negative colXα1 staining as no signal was observed.

### ColXα1 predicts NAC response in ER+/HER2+ cancer independently

We performed univariate and multivariate analyses using a logistic regression model in order to assess the associations between response, TILs, colXα1 IHC and other established clinicopathological parameters. Univariate analysis showed that high levels of colXα1 measured by IHC were associated with patients not achieving pCR or RCB I (OR = 18.88, *P* = 0.003) (Table 2). No patients with tumors with colXα1 scores of 2 or 3 achieved pCR or RCB I. More abundant stroma (OR = 6.92, *P* = 0.003) and positive lymph nodes (OR = 12.3, *P* = 0.003) were also associated with patients not achieving pCR or RCB I. In contrast, higher levels of TIL were associated with patients achieving good response (OR = 0.94, *P* = 0.001). Because the modest size of this study, multivariate analysis was performed comparing two variables at a time to avoid overfitting (Table 2).

**Fig. 4:**
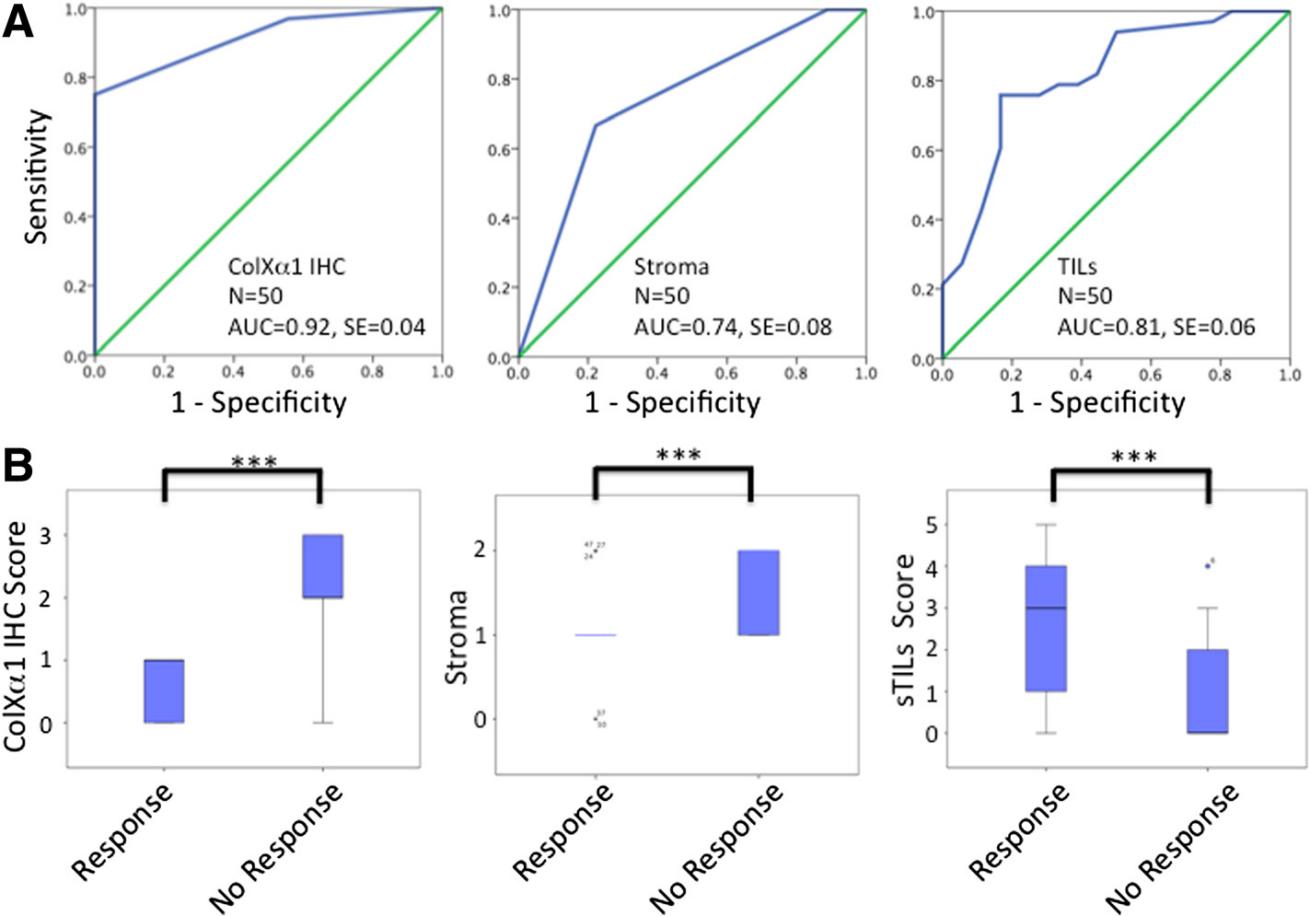
ColXα1 IHC scoring is strongly associated with NAC response. **a** ROC analysis of colXα1 IHC scores, stroma scores, and percent TIL. AUC = Area Under the Curve, SE = Standard Error. **b** Stroma and sTIL scores did not distinguish responders as strongly as colXα1 IHC. Box and whisker plots of each parameter show distinct separation between tumors that responded to NAC and those that with no response. *P < 0.05, ***P < 0.001

Multiple lines of evidence suggest that colXα1 IHC is a strong candidate marker. ColXα1 IHC discriminates good from poor responding patients with a low false positive rate. This is also reflected in the ROC curves where the colXα1 IHC is a more specific and sensitive marker of good response compared to stroma (Fig. 4a). ROC curves and box plots demonstrate that colXα1 and TIL strongly separate patients by good response, while the stroma score did not (Fig. 4). This indicated that high colXα1 expression by itself is an independent predictive factor, and not merely a reflection of more tumor associated stroma. Clinical biomarkers need to have very high specificity and sensitivity [20]. The high sensitivity, specificity, and accuracy of the colXα1 scoring support its further development as a marker for response in the NAC setting.

**Fig. 5:**
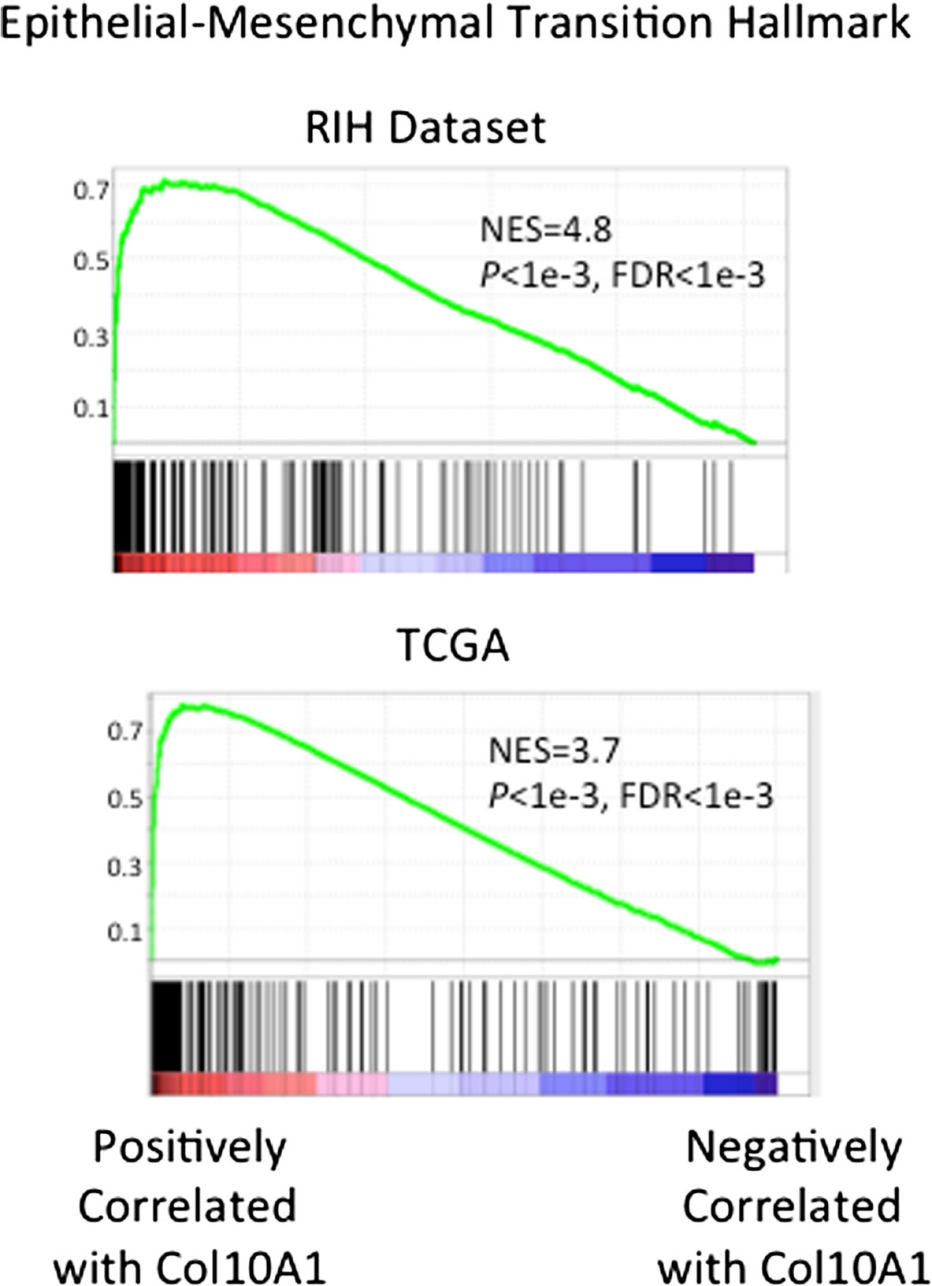
Col10A1 expression is correlated with epithelial-mesenchymal transition gene sets. GSEA reveals the Epithelial-Mesenchymal Transition Hallmark gene set is strongly enriched in Col10A1 positively correlated genes in both the RIH dataset and in 123 TCGA ER+/HER2+ breast tumors

To assess how colXα1 may be inducing chemoresistance, we evaluated the genes correlated with ColXA1 mRNA expression. Pathways associated with increased metabolism, chemoresistance and oncogenicity are strongly correlated with ColXA1 expression (see Additional file 5: Table S4). GSEA analysis of the ranked list of ColXA1 correlated genes revealed that the Epithelial-Mesenchymal Transition (EMT) hallmark gene set was strongly enriched (Fig. 5). Collagens are reported to help drive the mesenchymal state in tumors [21]. To further test the potential connection between ColXA1 and EMT we evaluated the co-expression in 123 TCGA invasive ER+/HER2+ breast tumors with RNA-seq expression data. Collagen positively correlated transcripts include EMT enrichment including the EMT transcription factor, SNAI2, GPX8, thought to help protect cells from oxidative damage, other collagens (Col12A1 and Col11A1) and collagen binding proteins including fibronectin, suggesting a broad network of a ColXA1 based network (see Additional file 5: Table S4). Collagens are known to increase matrix stiffness, which can induce EMT [22]. Other pathways enriched in highly correlated Col10A1 genes include TGFB signaling (TGFB3 and MAPK3). These findings support a connection between the expression of colXa1, EMT, the expression of putative resistance mechanisms, and response in the neoadjuvant setting.

## Discussion

The diversity of breast carcinoma is increasingly reflected in the spectrum of therapeutic approaches that are based on known biomarkers. ER, PR and HER2 are routinely used in clinical practice as a guide for the selection of therapy for breast cancer patients. In patients with stage II-III breast cancer, achievement of pCR to neoadjuvant chemotherapy correlates with improved long-term outcomes, however the predictive value of the standard clinical biomarkers such as ER, PR and HER2 is limited, motivating this study to identify factors mediating response. We defined the subtype of breast cancer by treatment protocols, and not the molecular markers used to define luminal vs. basal tumors [23]. We focused on the ER+/HER2+ subtype where less than 50 % of tumors respond to NAC (Table 1), despite combining HER2 targeted therapy, trastuzumab, with taxane and platinum based chemotherapy. To identify new markers, we combined RNA expression profiling with IHC to discover the importance of colXα1 positive stroma. Here, we found that collagens, namely colXα1, are up-regulated in breast tumors that do not respond to therapy in the ER+/HER2+ subtype. We observed an association at the mRNA level by qPCR and microarray and evaluated 50 cases at the protein level by IHC. Together, these RNA and protein data support the conclusion that colXα1 expression is strongly associated with chemotherapy response. We decided to focus on the role of collagens as collagens have been reported in gene expression signatures associated with response in the NAC setting and survival in the adjuvant setting. To our knowledge, this is the first study to evaluate the expression of colXα1 protein in breast tumors.

ColXα1 expression levels in the stroma of ER+/HER2+ tumors have a bimodal distribution, an important characteristic for a biomarker. While some ER+/HER2+ tumors do not express colXα1, those ER+/HER2+ tumors with strong expression (IHC score of 2 or 3) all were resistant to treatment. Thus, in this cohort, colXα1 predicts no false positives, and just 8 false negatives (Table 1).

The importance of the tumor microenvironment in influencing chemosensitivity is becoming increasingly clear [23]. The amount of stroma has been associated with chemosensitivity in many studies [24, 25]. Various stromal markers including tenascin, fibronectin and collagen type IV have been correlated with more aggressive behavior in breast cancer [26]. Although the quantity of stroma is correlated with pCR in this study, it is also clear that there are different types of stroma [27]. A variety of collagens are highly expressed in breast tumors contributing to its dense structure [9]. Collagens have long been known to be critical players in the extracellular matrix of breast tumors [9, 27], including mediating drug resistance [28], and alignment of collagens has been proposed to indicate progression in breast tumors [29, 30]. Collagen alignment was reported to correlate with expression of syndecan-1, but this gene was not significantly differentially expressed in this study (Fc = 0.15). However, there are limited data on the expression and function of many specific collagen subtypes by IHC in breast cancer patients. Collagen is associated with the major ECM transformations and the collagen subtype COLl11A1 has been associated with metastasis and disease progression in breast cancer [9]. In this study, COL3A1 and COL14A1 are among the most significantly differentially expressed transcripts (see Additional file 4: Table S3) and are good candidates for further evaluation, as they are not one of the dominant forms of collagen in normal breast tissue. Further studies are warranted to test which collagens, and if a combination of specific collagens are good predictors of response at the RNA and protein levels.

Col10A1 mRNA expression is up-regulated in a variety of human malignancies compared to normal tissue, including breast tumors [8]. Increased expression of Col10A1 has been a part of breast cancer signatures, including a CD10+ signature to discriminate in situ from invasive breast cancer [11] and a stroma expression signature to predict resistance to neoadjuvant chemotherapy in breast cancer [10], though the specific ER+/HER2+ subtype was not specifically evaluated. In normal tissue, colXα1 expression is distinct among all collagens as it is only expressed in hypertrophic chondrocytes [7]. These observations and our data highlight how colXα1 can be an excellent biomarker for chemoresistance and is a candidate target for specific delivery to ER+/HER2+ breast tumors. It is not clear why expression of the COL10A1 gene is such a good marker. In pre-clinical models, collagens increase multiple tumor properties including growth, tumorigenicity, invasion [9, 31], the drug resistance, and the mesenchymal state [32, 33]. Here, many related genes and pathways involved in chemoresistance were correlated with COL10A1 mRNA expression. These observations further support the development and testing of colXa1 protein expression as a biomarker, perhaps in combination with other pathways such as sTILs, EMT markers. Further study is warranted to determine the function of colXα1 in mediating resistance.

We were motivated to look at TILs because of the enrichment of immune pathways, including genes indicative of T cells, in the gene expression data. Several clinical studies have evaluated TILs as a positive prognostic biomarker in TNBC [2, 34–42]. However, TILs were not a positive biomarker for luminal subtypes [43]. In this study, TILs were a strong independent predictor in in ER+/HER2+ subtypes (Table 2, see Additional file 7: Table S5). In the ER+/HER2+ subtype, TILs and colXα1 both contribute to predict chemosensitivity (Fig. 4, Tables 1 and 2), suggesting that the combination of TILs and colXα1 IHC score is a strong predictor for ER+/HER+ breast cancers.

This study focused on patients with ER+/HER2+ breast cancer treated with neoadjuvant chemotherapy and HER2-targeted therapy and was designed to identify novel biomarkers predictive of pathologic response. This definition may have impacted our ability to detect Col10A1 and TIL association not previously observed in luminal tumors. The HER2-positive patients generally have a significantly higher pCR rate in response to NAC+H. However, within the HER2-positive population, pCR was more common for ER-negative tumors than for ER- positive tumors [4, 5, 44]. This suggests that for a subset of HER2+ tumors, ER or a more complex molecular pathway drives response, and ER+/HER2+ tumors are biologically different than ER-/HER2+ tumors [45, 46].

Our data represent initial, preliminary evidence that colXα1 may be a marker for NAC response. The present study has some limitations, for example, the modest number of cases could be affected by unknown sample biases. We have selected cases with similar, but not identical treatment schedules. Even with a variety of treatment schedules, TILs and colXα1 have strong predictive power for chemoresponse suggesting that they are general factors for NAC responsiveness in ER+/HER2+ breast tumors and further study of colXα1 and other collagens as predictive markers is warranted.

## Conclusions

In summary, this is the first description, to our knowledge, of an association between a specific collagen subtype and response to neoadjuvant chemotherapy in breast cancer. Although our data cannot confirm causality, the significant difference in colXα1 expression in the responsive and non-responsive tumors, are striking. Together with the many studies showing that collagens, as part of the extracellular matrix, induce significant differences in the differentiation and life/death promotion of cancer cells, this study suggests that stromal expressed colXα1 plays a causative role in the tumor’s response to therapy. The evaluation of colXα1 protein levels provides a robust marker in predicting responses and warrants further evaluation in larger studies.

## Availability of data and materials

The microarray data have been deposited into GEO, GSE67982.

## Additional files

Additional file 1: Table S1. Patient characteristics. x indicates the case was included for the assay. (XLSX 44 kb) Additional file 2: Table S2. List of 30 genes significantly differentially expressed between good and poor responders. Rows in blue highlight the collagens. (XLSX 53 kb) Additional file 3: Figure S1. qPCR validation of Affymetrix microarray of Col10A1, DACH1, RAB32, STC2, and FASN. Expression was calculated relative to GAPDH. (PPTX 51 kb) Additional file 4: Table S3. Gene Ontology (GO) gene sets enriched in either good or poor responding  tumors. The gene sets further examined in this study are highlighted in blue. (XLSX 70 kb) Additional file 5: Table S4. Cancer hallmarks gene sets enriched in positively or negatively correlated Col10A1 expression. (XLSX 17 kb) Additional file 6: Figure S2. 20× image of the H&E stained RCB3 case shown in Fig. 3 to highlight the number of tumor cells in this case. (PNG 289 kb) Additional file 7: Table S5. Univariate and multivariate logistic regression for prediction of pCR in ER+/HER2 *N* = 74. (DOCX 53 kb)

## Abbreviations

EFS: event-free survival
GO: gene ontology
GSEA: Gene set enrichment analysis
IHC: immunohistochemistry
NAC: neoadjuvant chemotherapy
NAC+H: NEOadjuvant chemotherapy and HER2-targeted therapy
OR: odds ratio
pCR: pathological complete response
RCB: residual cancer burden
ROC: receiver operator characteristic
SE: standard error
sTILs: stroma tumor infiltrating lymphocytes
TILs: tumor infiltrating lymphocytes
TNBC: triple negative breast cancer
