# Supplementary material for: Identification of stromal ColXα1 and tumor-infiltrating lymphocytes as putative predictive markers of neoadjuvant therapy in estrogen receptor-positive/HER2-positive breast cancer
Source: BMC Cancer. 2016 Apr 18;16:274. doi: 10.1186/s12885-016-2302-5 (PMC4835834; doi:10.1186/s12885-016-2302-5)
Supplement: Additional file 3: Figure S1. — qPCR validation of Affymetrix microarray of Col10A1, DACH1, RAB32, STC2, and FASN. Expression was calculated relative to GAPDH. (PPTX 51 kb) [file 12885_2016_2302_MOESM3_ESM.pptx]

## Slide 1
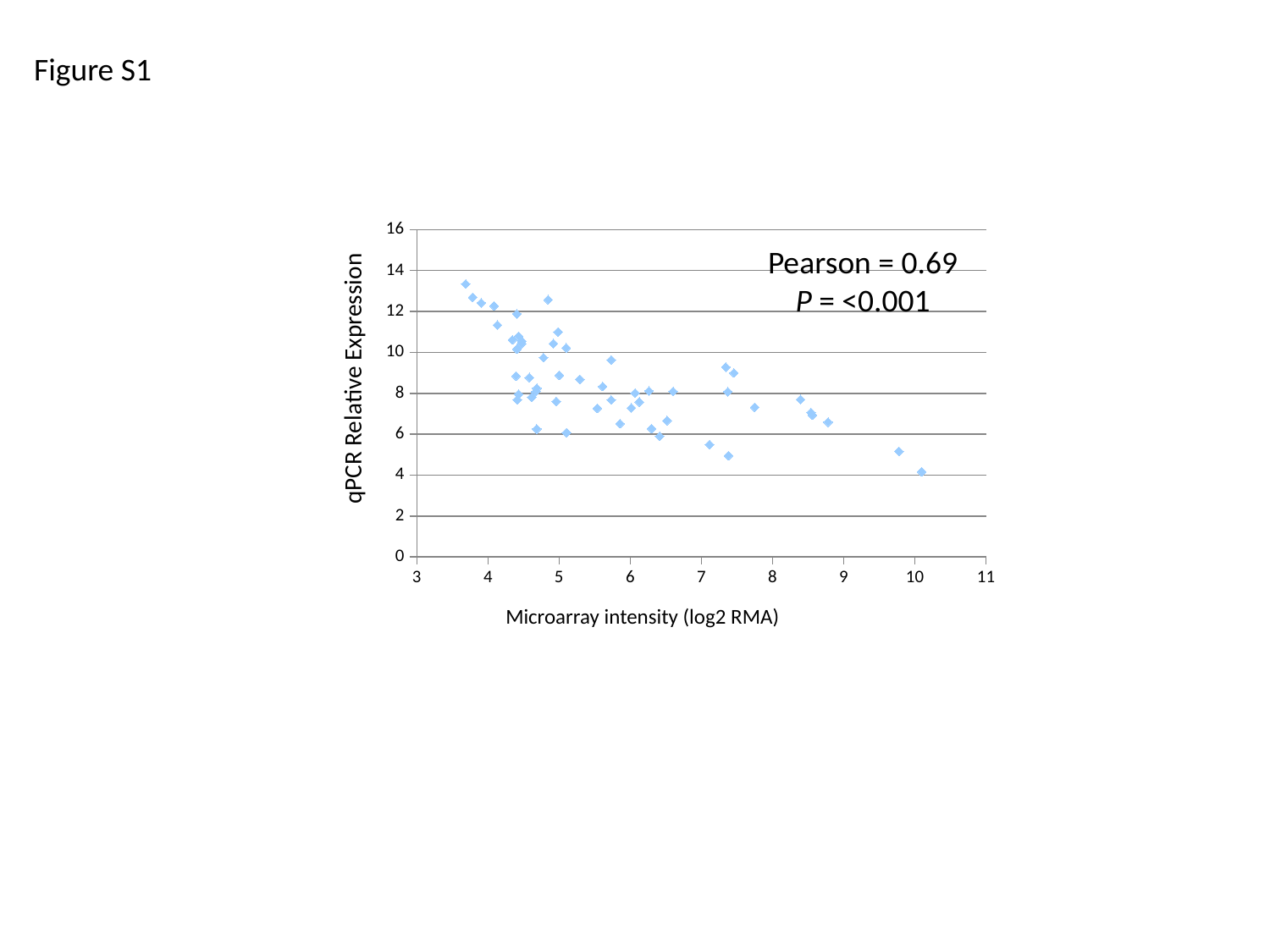

Figure S1
### Chart
| Category | |
|---|---|Pearson = 0.69
P = <0.001
qPCR Relative Expression
Microarray intensity (log2 RMA)
