# Supplementary figures and images for: Identification of stromal ColXα1 and tumor-infiltrating lymphocytes as putative predictive markers of neoadjuvant therapy in estrogen receptor-positive/HER2-positive breast cancer
Source: BMC Cancer. 2016 Apr 18;16:274. doi: 10.1186/s12885-016-2302-5 (PMC4835834; doi:10.1186/s12885-016-2302-5)

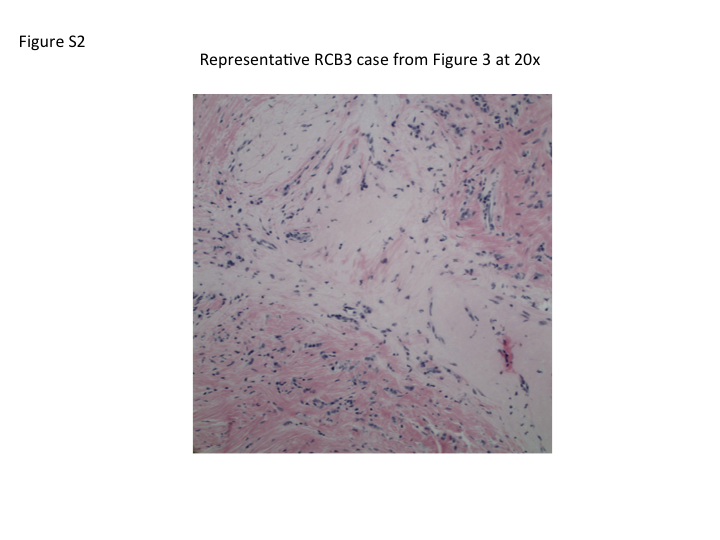

Supplement: Additional file 6: Figure S2. — 20× image of the H&E stained RCB3 case shown in Fig. 3 to highlight the number of tumor cells in this case. (PNG 289 kb) [file 12885_2016_2302_MOESM6_ESM.png]
