# Supplementary material for: Identification of stromal ColXα1 and tumor-infiltrating lymphocytes as putative predictive markers of neoadjuvant therapy in estrogen receptor-positive/HER2-positive breast cancer
Source: BMC Cancer. 2016 Apr 18;16:274. doi: 10.1186/s12885-016-2302-5 (PMC4835834; doi:10.1186/s12885-016-2302-5)
Supplement: Additional file 7: Table S5. — Univariate and multivariate logistic regression for prediction of pCR in ER+/HER2 N = 74. (DOCX 53 kb) [file 12885_2016_2302_MOESM7_ESM.docx]

**Table S5. Univariate and multivariate logistic regression for prediction of pCR in ER+/HER2**

**N=74.**

Univariate

|  | HR (95% CI) | *P* value |
| --- | --- | --- |
| Age | 1.04 (1.003 – 1.08) | 0.04 |
| TIL | 0.93 (0.9 – 0.96) | <1e-3 |
| Stroma | 9.3 (3.0 – 28.7) | <1e-3 |

Multivariate

| Age + stroma | Age | 1.03 (0.99 – 1.07) | 0.14 |
| --- | --- | --- | --- |
|  | stroma | 8.5 (2.7 – 26.3) | <1e-3 |
|  |  |  |  |
| Age + TIL | Age | 1.02 (0.97 – 1.07) | 0.44 |
|  | TIL | 0.93 ( 0.91 – 0.96) | <1e-3 |
|  |  |  |  |
| TIL + Stroma | TIL | 0.93 (0.9 – 0.97) | <1e-3 |
|  | stroma | 9.8 (2.1 – 44.3) | 0.003 |
